# Aubergine and Potato Sensitivity with Latex Sensitisation and Oral Allergy Syndrome

**DOI:** 10.1155/2013/314658

**Published:** 2013-07-11

**Authors:** A. S. Bansal

**Affiliations:** Department of Immunology and Allergy, St. Helier Hospital, Carshalton, Surrey SM5 1AA, UK

## Abstract

Aubergine allergy is rare outside of India and the far east, and very few cases have been reported. We describe a case of aubergine allergy in a 9-year-old girl of Anglo-Indian descent who also had sensitivity to potato, coincidental oral allergy syndrome, and latex sensitisation with mild oral symptoms on consuming kiwi fruit. Specific IgE to aubergine was negative, but skin testing was positive to both raw and cooked aubergine. With early and increased consumption of exotic vegetables in western countries, more cases of aubergine allergy can be expected and negative blood tests do not exclude type 1 sensitivity.

## 1. Introduction

The literature on aubergine allergy suggests that it is very rare outside of India and Spain. While this may be related to early age and frequency of exposure, the precise reasons for this are unclear. We report a case of aubergine allergy in a young girl who also had sensitivity to potato, symptoms of an oral allergy syndrome, and latex sensitization and in whom skin prick tests were confirmatory, but the blood tests were essentially negative. To the best of my knowledge, this is the first report of this combination of allergies. 

## 2. Case Report

A 9-year-old girl of Anglo-Indian descent developed oral itching and significant perioral urticaria after consuming 20 gm of cooked aubergine. She had previously suffered facial swelling after peeling potatoes, but eating cooked potatoes did not cause any symptoms. Antihistamines were helpful for these reactions and there was no cardiorespiratory compromise or gastrointestinal upset. In the past, she had noted oral itching on consuming fresh apples and pears. Other fruits, cooked apples, and tree nuts were tolerated. The patient had mild hay fever and eczema but no asthma. There is no significant past medical history. There was a family history of atopy in both parents in the form of asthma and hay fever.

Skin prick testing (SPT) confirmed 6 mm wheals to potato and mixed grass and silver birch pollen. SPT of fresh and cooked aubergine was performed using material just beneath the skin to capture all the important aubergine allergens as suggested by Babu and Venkatesh [1]. This revealed 5 mm wheals to both. A 3 mm wheal was evident to hazel nut, fresh apple, and house dust mite. Negative responses were returned to weed and shrub pollen as well as to fresh pear and plum. The histamine positive control at 1 mg/mL returned a 5 mm weal while it was negative when used at 0.1 mg/mL. Consumption of 0.5 gm of cooked aubergine in the clinic reproduced oral itching but did not induce a skin rash. The challenge was ceased at this point. 

The results of the patient's blood tests performed on the Immunocap analyser (Thermo-Fisher, UK) are detailed in Table 1. The significant levels of specific IgE to the BetV1 protein and to apple confirmed the patient's oral allergy syndrome (OAS). However, the addition of a saline extract made from fresh aubergine to the patient's serum prior to retesting for BetV1 specific IgE showed less than 5% reduction from 55.9 to 53.5 kU_A_/L. The specific IgE to aubergine was negative according to the adult range but possibly positive according to the paediatric range. The negative specific IgE to potato with a positive result on SPT is in keeping with the OAS. The negative result to bromelain makes it very unlikely that the patient's various positive results are due to cross reactive carbohydrate determinants in someone with latex sensitisation. 

## 3. Discussion

Of the 740 randomly selected subjects reported by Babu et al. [2] from Mysore in India, 9.2% reported adverse reactions to eating aubergine and of these just under a half (4.3%) returned a positive history and a positive SPT. True aubergine allergy was more frequent in females and in those with atopy. In common with our own finding, only 6 of the 48 SPT-positive subjects had demonstrable specific IgE to aubergine. 

Aubergine has previously been considered to produce nonspecific SPT results owing to its significant histamine content [3]. This was reported to be 0.89 mg/100 gm for the round purple aubergine which decreased slightly by 10%–14% on cooking. Furthermore, SPT in nonatopic subjects was negative in the majority of individuals unless there was high sensitivity to histamine with reactivity to 10–100 *μ*g/mL. In our patient sensitivity to histamine was unlikely to explain her positive response to aubergine as there was no response to the histamine control used at 0.1 mg/mL. Whether the sensitivity evident in our patient is due to the 1 kD nonprotein metabolite evident on SPT by Pramod and Venkatesh [4] after their size-exclusion chromatography of the 10 kd filtrate of eggplant extract on Sephadex G-25 is unclear. 

Our patient's combined potato and aubergine sensitivity raised the possibility of a Solanaceae sensitivity. While rare, cross-reactivity between aubergine and vegetables that are members of the Solanceae family has previously been suggested [5]. Thankfully, our patient's sensitivity to potato was restricted to local reactivity on cutaneous contact with raw peeled potatoes and eating cooked potatoes did not cause any symptoms. This would suggest that the potato sensitivity was more likely to be part of the OAS. In this regard the lack of inhibition of the patient's specific IgE to the BetV1 protein by the saline extract of fresh aubergine confirms that the aubergine allergy was separate from the patient's OAS and the two are likely coincidental. Regardless, the combination of aubergine allergy and OAS has rarely been reported in the past. This may be because the vast majority of reports on aubergine allergy have emanated from India where OAS is rare owing to few Silver birch trees. As a general point the BetV1 homologous proteins that form the basis of the OAS are fragile and do not retain their allergenic structure within commercial skin testing reagents. As such prick-to-prick testing with fresh raw vegetables and fruit is more likely to be positive and is a more sensitive and reproducible technique for investigating suspected fruit and vegetable allergy. However, this testing confirms only a broad pattern of sensitivity and is not necessarily specific for the individual type of fruit and vegetable.

Aubergine allergy has previously been demonstrated in association with latex allergy [6, 7]. In consequence this raises the possibility that it may also be linked with the latex/tropical fruit allergy syndrome or latex/vegetable syndrome. In the present case there was no evidence of reactivity to the rHevb5 or rHevb6.02 with borderline sensitivity to latex and avocado by specific IgE antibody testing but in the absence of clinical reactivity. While it is possible that the patient's sensitivity to latex and the aubergine are the result of a general profilin sensitivity, this is unlikely as the result to the Hevb8 was negative. Interestingly, significant IgE antibodies were evident to chestnuts although these had not been consumed previously. Furthermore, there was significant sensitivity to the kiwi fruit rActd8 protein and the patient subsequently mentioned mild oral tingling on consuming this fruit. It is of course likely that the Actd8 sensitivity is related to the fact that this is a recognised BetV1 homologous protein [8].

In conclusion aubergine allergy remains rare in the Western world but if suspected it may require skin testing for confirmation as specific IgE testing may be erroneous. The precise factor(s) within aubergine which mediates this sensitivity is unclear.

## Figures and Tables

**Table 1:** Serum specific IgE results to relevant allergens.

Food	Specific IgE (kU_A_/l)	Recombinant food protein	Specific IgE (kU_A_/l)
Apple	4.7	Bet V1	55.9
Aubergine	0.21	rHev b5	<0.35
Avocado	0.35	rHev b6.02	<0.35
Chestnut	1.8	rHev b8	0.23
Kiwi	0.66	rAct d8	17.0
Latex	0.36	Bromelain	<0.1
Pineapple	<0.1	Pru p3 peach LTP	<0.1
Potato	<0.1		
Tomato	0.14		
